# Dibromidobis[1-(2-bromo­benz­yl)-3-(pyrimidin-2-yl)-1*H*-imidazol-2(3*H*)-one]copper(II)

**DOI:** 10.1107/S1600536812021460

**Published:** 2012-05-19

**Authors:** Chun-Xin Lu

**Affiliations:** a Department of Chemistry, Zhejiang University, Xixi Campus, Hangzhou 310028, People’s Republic of China

## Abstract

In the title complex, [CuBr_2_(C_14_H_11_BrN_4_O)_2_], the Cu^II^ ion is located on an inversion centre and is coordinated by two ketonic O atoms, two N atoms and two Br atoms, forming a distorted octahedral coordination environment. The two carbonyl groups are *trans* positioned with C=O bond lengths of 1.256 (5) Å, in agreement with a classical carbonyl bond. The Cu—O bond length is 2.011 (3) Å. The two bromo­benzyl rings are approximately parallel to one another, forming a dihedral angle of 70.1 (4)° with the coordination plane.

## Related literature

For general background, see: Moncol *et al.* (2008 ▶); Wu *et al.* (2003 ▶); Anbu & Kandaswamy (2012 ▶). For related structures, see: Citadelle *et al.* (2010 ▶); Liu *et al.* (2011 ▶); Marjani *et al.* (2005 ▶); Meghdadi *et al.* (2012 ▶).
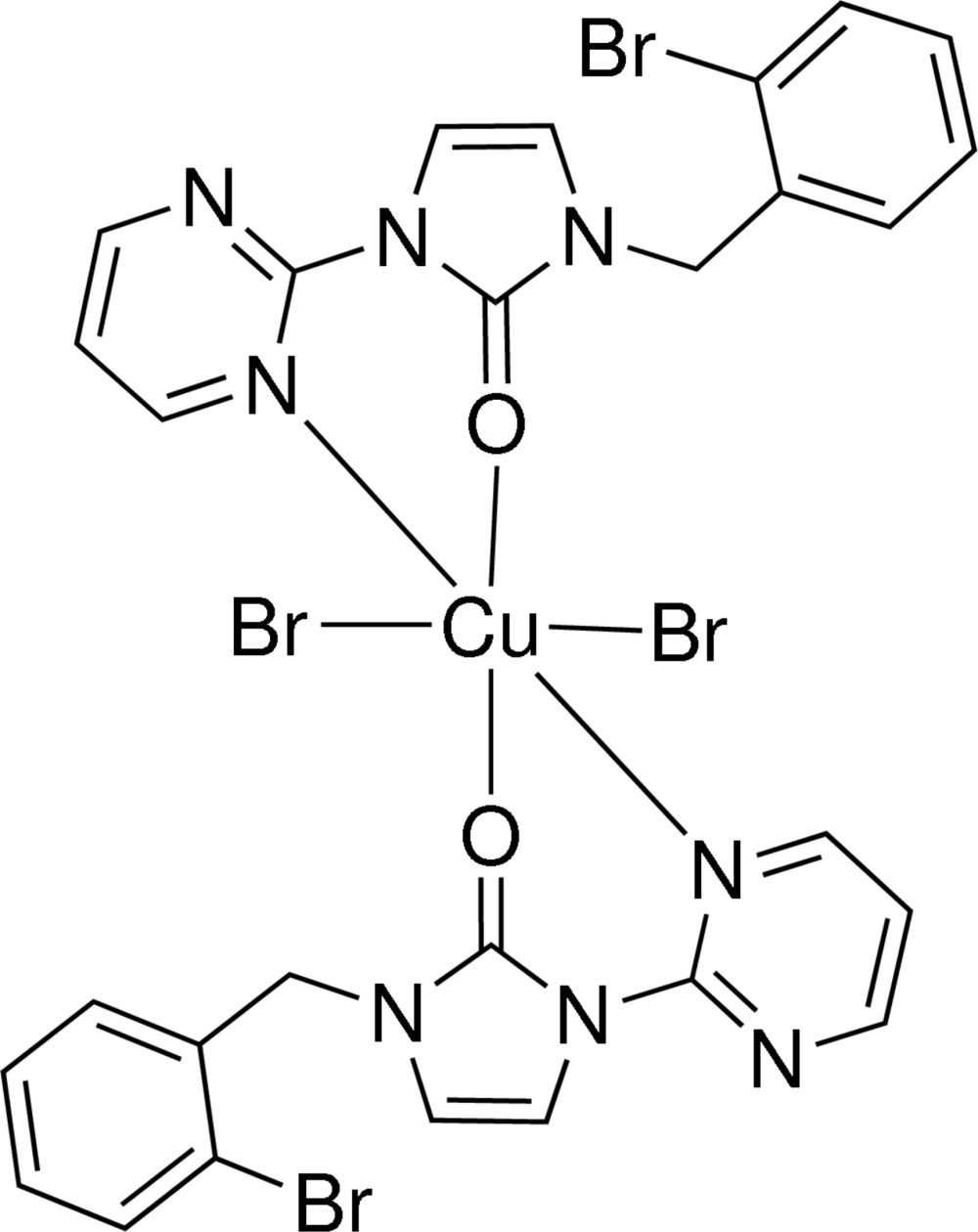


## Experimental

### 

#### Crystal data

[CuBr_2_(C_14_H_11_BrN_4_O)_2_]
*M* *_r_* = 885.72
Monoclinic, 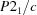

*a* = 8.6803 (11) Å
*b* = 23.0354 (8) Å
*c* = 7.8543 (9) Å
β = 109.419 (1)°
*V* = 1481.2 (3) Å^3^
*Z* = 2
Mo *K*α radiation
μ = 6.18 mm^−1^
*T* = 298 K
0.43 × 0.30 × 0.14 mm

#### Data collection

Bruker SMART CCD area-detector diffractometer
Absorption correction: multi-scan (*SADABS*; Sheldrick, 1996 ▶) *T*_min_ = 0.177, *T*_max_ = 0.479
7275 measured reflections
2622 independent reflections
2019 reflections with *I* > 2σ(*I*)
*R*_int_ = 0.066

#### Refinement

*R*[*F* ^2^ > 2σ(*F* ^2^)] = 0.045
*wR*(*F* ^2^) = 0.117
*S* = 1.01
2622 reflections
196 parameters
H-atom parameters constrained
Δρ_max_ = 1.31 e Å^−3^
Δρ_min_ = −0.90 e Å^−3^

### 

Data collection: *SMART* (Bruker, 2002 ▶); cell refinement: *SAINT* (Bruker, 2002 ▶); data reduction: *SAINT*; program(s) used to solve structure: *SHELXTL* (Sheldrick, 2008 ▶); program(s) used to refine structure: *SHELXTL*; molecular graphics: *SHELXTL*; software used to prepare material for publication: *SHELXTL*.

## Supplementary Material

Crystal structure: contains datablock(s) global, I. DOI: 10.1107/S1600536812021460/ru2034sup1.cif

Supplementary material file. DOI: 10.1107/S1600536812021460/ru2034Isup2.cdx

Structure factors: contains datablock(s) I. DOI: 10.1107/S1600536812021460/ru2034Isup2.hkl

Additional supplementary materials:  crystallographic information; 3D view; checkCIF report

## Figures and Tables

**Fig. 1.**
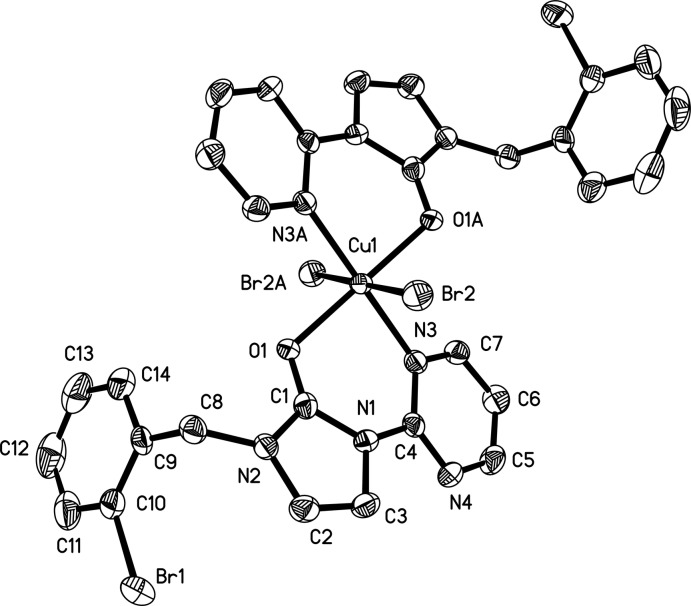
The molecular structure of the title compound, showing 50% probability displacement ellipsoids and the atomic numbering.

## supplementary crystallographic information

### Comment

Cu^2+^ cation has been widely studied since a host of low-molecular-weight copper complexes have been proven beneficial against several diseases such as turberculosis, rheumatoid, gastric ulcers, and cancers. And it is well known that copper(II) complexes with different ligands usually show flexible coordination environment. The 1-(2-bromobenzyl)-3-(pyrimidin-2-yl)imidazolium bromide was used as the ligand, reacting with excessive copper powder in air, giving a Cu^II^ compound. We here report the crystal structure of the title compound (I).

Bond lengths and angles in the title molecule (Fig. 1) are within normal ranges. The C=O bond distance is 1.256 (5) Å and Cu—O bond distance is 2.013 (3) Å. The two bromobenzyl rings are approximately parallel to each other. The dihedral angle between the bromobenzyl ring and the coordination plane is 70.1 (4)°.

### Experimental

A solution of 1-(2-bromobenzyl)-3-(pyrimidin-2-yl)imidazolium bromide (396 mg, 1.0 mmol) in 10 ml of CH_3_CN was treated with copper powder (38 mg, 0.6 mmol). The mixture was allowed to react at 80 °C for 2 days in air. The solution was filtered through silica to remove unreacted copper. The filtrate was concentrated to *ca* 2 ml. Addition of Et_2_O (20 ml) to the filtrate afforded a yellow precipitate. The crystals of this complex suitable for X-ray diffraction were obtained by slow diffusion of diethyl ether into its acetonitrile solution.

### Refinement

H atoms were placed in calculated positions with C—H = 0.93–0.97 Å, and refined in riding mode with *U*_iso_(H) = 1.2*U*_eq_(C).

### Crystal data

**Table:**

[CuBr_2_(C_14_H_11_BrN_4_O)_2_]	*F*(000) = 862
*M**_r_* = 885.72	*D*_x_ = 1.986 Mg m^−^^3^
Monoclinic, *P*2_1_/*c*	Mo *K*α radiation, λ = 0.71073 Å
*a* = 8.6803 (11) Å	Cell parameters from 7275 reflections
*b* = 23.0354 (8) Å	θ = 1.8–25.1°
*c* = 7.8543 (9) Å	µ = 6.18 mm^−^^1^
β = 109.419 (1)°	*T* = 298 K
*V* = 1481.2 (3) Å^3^	Block, yellow
*Z* = 2	0.43 × 0.30 × 0.14 mm

### Data collection

**Table:**

Bruker SMART CCD area-detector diffractometer	2622 independent reflections
Radiation source: fine-focus sealed tube	2019 reflections with *I* > 2σ(*I*)
Graphite monochromator	*R*_int_ = 0.066
phi and ω scans	θ_max_ = 25.1°, θ_min_ = 1.8°
Absorption correction: multi-scan (*SADABS*; Sheldrick, 1996)	*h* = −10→8
*T*_min_ = 0.177, *T*_max_ = 0.479	*k* = −27→20
7275 measured reflections	*l* = −9→8

### Refinement

**Table:**

Refinement on *F*^2^	Primary atom site location: structure-invariant direct methods
Least-squares matrix: full	Secondary atom site location: difference Fourier map
*R*[*F*^2^ > 2σ(*F*^2^)] = 0.045	Hydrogen site location: inferred from neighbouring sites
*wR*(*F*^2^) = 0.117	H-atom parameters constrained
*S* = 1.01	*w* = 1/[σ^2^(*F*_o_^2^) + (0.0654*P*)^2^] where *P* = (*F*_o_^2^ + 2*F*_c_^2^)/3
2622 reflections	(Δ/σ)_max_ < 0.001
196 parameters	Δρ_max_ = 1.31 e Å^−^^3^
0 restraints	Δρ_min_ = −0.90 e Å^−^^3^

### Special details

**Table:**

Geometry. All e.s.d.'s (except the e.s.d. in the dihedral angle between two l.s. planes) are estimated using the full covariance matrix. The cell e.s.d.'s are taken into account individually in the estimation of e.s.d.'s in distances, angles and torsion angles; correlations between e.s.d.'s in cell parameters are only used when they are defined by crystal symmetry. An approximate (isotropic) treatment of cell e.s.d.'s is used for estimating e.s.d.'s involving l.s. planes.
Refinement. Refinement of *F*^2^ against ALL reflections. The weighted *R*-factor *wR* and goodness of fit *S* are based on *F*^2^, conventional *R*-factors *R* are based on *F*, with *F* set to zero for negative *F*^2^. The threshold expression of *F*^2^ > σ(*F*^2^) is used only for calculating *R*-factors(gt) *etc*. and is not relevant to the choice of reflections for refinement. *R*-factors based on *F*^2^ are statistically about twice as large as those based on *F*, and *R*- factors based on ALL data will be even larger.

### Fractional atomic coordinates and isotropic or equivalent isotropic displacement parameters (Å^2^)

**Table:**

	*x*	*y*	*z*	*U*_iso_*/*U*_eq_	
Cu1	0.5000	0.5000	0.5000	0.0270 (3)	
N1	0.7847 (4)	0.41135 (18)	0.7064 (5)	0.0240 (9)	
N2	0.6658 (4)	0.37501 (18)	0.8889 (5)	0.0245 (9)	
N3	0.6973 (5)	0.46886 (18)	0.4448 (5)	0.0240 (9)	
N4	0.9437 (5)	0.41415 (19)	0.5252 (6)	0.0307 (11)	
Br1	0.68599 (7)	0.22945 (3)	1.05716 (9)	0.0525 (2)	
Br2	0.70578 (6)	0.54952 (2)	0.82796 (7)	0.0343 (2)	
O1	0.5017 (4)	0.42702 (15)	0.6410 (4)	0.0271 (8)	
C1	0.6372 (5)	0.4069 (2)	0.7368 (6)	0.0249 (11)	
C2	0.8315 (6)	0.3607 (2)	0.9542 (7)	0.0308 (13)	
H2	0.8821	0.3393	1.0582	0.037*	
C3	0.9052 (6)	0.3822 (2)	0.8467 (7)	0.0300 (12)	
H3	1.0154	0.3788	0.8603	0.036*	
C4	0.8100 (6)	0.4324 (2)	0.5504 (7)	0.0242 (11)	
C5	0.9699 (6)	0.4335 (2)	0.3762 (8)	0.0372 (14)	
H5	1.0654	0.4226	0.3557	0.045*	
C6	0.8598 (6)	0.4692 (2)	0.2517 (7)	0.0342 (13)	
H6	0.8773	0.4814	0.1467	0.041*	
C7	0.7231 (6)	0.4854 (2)	0.2919 (7)	0.0286 (12)	
H7	0.6457	0.5088	0.2104	0.034*	
C8	0.5415 (6)	0.3582 (2)	0.9661 (7)	0.0295 (12)	
H8A	0.4736	0.3916	0.9664	0.035*	
H8B	0.5946	0.3464	1.0905	0.035*	
C9	0.4349 (6)	0.3096 (2)	0.8659 (7)	0.0295 (12)	
C10	0.4763 (6)	0.2516 (3)	0.8917 (7)	0.0344 (13)	
C11	0.3759 (8)	0.2078 (3)	0.8035 (9)	0.0502 (17)	
H11	0.4089	0.1693	0.8262	0.060*	
C12	0.2274 (8)	0.2208 (3)	0.6820 (10)	0.058 (2)	
H12	0.1585	0.1911	0.6207	0.070*	
C13	0.1780 (7)	0.2780 (3)	0.6490 (9)	0.0559 (19)	
H13	0.0767	0.2868	0.5648	0.067*	
C14	0.2800 (6)	0.3221 (3)	0.7416 (8)	0.0414 (15)	
H14	0.2455	0.3605	0.7213	0.050*	

### Atomic displacement parameters (Å^2^)

**Table:**

	*U*^11^	*U*^22^	*U*^33^	*U*^12^	*U*^13^	*U*^23^
Cu1	0.0258 (4)	0.0231 (5)	0.0378 (5)	0.0072 (4)	0.0181 (4)	0.0100 (4)
N1	0.023 (2)	0.023 (2)	0.028 (2)	0.0032 (18)	0.0107 (17)	0.0006 (19)
N2	0.027 (2)	0.022 (2)	0.026 (2)	−0.0005 (19)	0.0111 (17)	0.0003 (19)
N3	0.027 (2)	0.021 (2)	0.025 (2)	0.0001 (19)	0.0106 (18)	0.0016 (19)
N4	0.026 (2)	0.032 (3)	0.040 (3)	0.004 (2)	0.0180 (19)	0.002 (2)
Br1	0.0517 (4)	0.0387 (4)	0.0715 (5)	0.0133 (3)	0.0262 (3)	0.0235 (3)
Br2	0.0355 (3)	0.0329 (4)	0.0373 (4)	0.0010 (2)	0.0158 (2)	−0.0018 (2)
O1	0.0232 (17)	0.0231 (19)	0.037 (2)	0.0054 (15)	0.0129 (15)	0.0114 (16)
C1	0.027 (3)	0.025 (3)	0.025 (3)	−0.002 (2)	0.011 (2)	0.000 (2)
C2	0.029 (3)	0.034 (3)	0.027 (3)	0.004 (2)	0.006 (2)	0.006 (2)
C3	0.023 (2)	0.035 (3)	0.032 (3)	0.005 (2)	0.008 (2)	0.004 (3)
C4	0.027 (2)	0.018 (3)	0.031 (3)	−0.003 (2)	0.014 (2)	−0.004 (2)
C5	0.033 (3)	0.037 (4)	0.050 (4)	−0.001 (3)	0.025 (3)	−0.007 (3)
C6	0.042 (3)	0.034 (3)	0.035 (3)	−0.001 (3)	0.023 (3)	−0.005 (3)
C7	0.029 (3)	0.025 (3)	0.032 (3)	0.001 (2)	0.010 (2)	−0.002 (2)
C8	0.037 (3)	0.028 (3)	0.029 (3)	0.003 (3)	0.017 (2)	0.003 (2)
C9	0.035 (3)	0.027 (3)	0.034 (3)	−0.002 (2)	0.021 (2)	0.007 (2)
C10	0.034 (3)	0.034 (3)	0.042 (3)	0.000 (3)	0.021 (2)	0.003 (3)
C11	0.058 (4)	0.034 (4)	0.074 (5)	−0.012 (3)	0.042 (4)	−0.008 (3)
C12	0.055 (4)	0.055 (5)	0.073 (5)	−0.030 (4)	0.033 (4)	−0.027 (4)
C13	0.033 (3)	0.078 (6)	0.056 (4)	−0.013 (4)	0.014 (3)	−0.002 (4)
C14	0.037 (3)	0.047 (4)	0.046 (3)	0.002 (3)	0.022 (3)	0.006 (3)

### Geometric parameters (Å, º)

**Table:**

Cu1—O1^i^	2.011 (3)	C3—H3	0.9300
Cu1—O1	2.011 (3)	C5—C6	1.387 (8)
Cu1—N3^i^	2.032 (4)	C5—H5	0.9300
Cu1—N3	2.032 (4)	C6—C7	1.378 (7)
Cu1—Br2	2.8404 (6)	C6—H6	0.9300
Cu1—Br2^i^	2.8404 (6)	C7—H7	0.9300
N1—C1	1.383 (6)	C8—C9	1.498 (7)
N1—C4	1.400 (6)	C8—H8A	0.9700
N1—C3	1.412 (6)	C8—H8B	0.9700
N2—C1	1.353 (6)	C9—C10	1.381 (8)
N2—C2	1.396 (6)	C9—C14	1.404 (7)
N2—C8	1.455 (6)	C10—C11	1.362 (8)
N3—C4	1.346 (6)	C11—C12	1.358 (9)
N3—C7	1.348 (6)	C11—H11	0.9300
N4—C4	1.309 (6)	C12—C13	1.384 (9)
N4—C5	1.340 (7)	C12—H12	0.9300
Br1—C10	1.921 (5)	C13—C14	1.384 (9)
O1—C1	1.256 (5)	C13—H13	0.9300
C2—C3	1.314 (7)	C14—H14	0.9300
C2—H2	0.9300		
			
O1^i^—Cu1—O1	180.00 (11)	N4—C4—N1	115.2 (4)
O1^i^—Cu1—N3^i^	88.28 (15)	N3—C4—N1	117.5 (4)
O1—Cu1—N3^i^	91.72 (15)	N4—C5—C6	122.4 (5)
O1^i^—Cu1—N3	91.72 (15)	N4—C5—H5	118.8
O1—Cu1—N3	88.28 (15)	C6—C5—H5	118.8
N3^i^—Cu1—N3	180.000 (1)	C7—C6—C5	116.3 (5)
O1^i^—Cu1—Br2	92.92 (10)	C7—C6—H6	121.9
O1—Cu1—Br2	87.08 (10)	C5—C6—H6	121.9
N3^i^—Cu1—Br2	89.16 (11)	N3—C7—C6	122.6 (5)
N3—Cu1—Br2	90.84 (11)	N3—C7—H7	118.7
O1^i^—Cu1—Br2^i^	87.08 (10)	C6—C7—H7	118.7
O1—Cu1—Br2^i^	92.92 (10)	N2—C8—C9	113.2 (4)
N3^i^—Cu1—Br2^i^	90.84 (11)	N2—C8—H8A	108.9
N3—Cu1—Br2^i^	89.16 (11)	C9—C8—H8A	108.9
Br2—Cu1—Br2^i^	180.00 (2)	N2—C8—H8B	108.9
C1—N1—C4	126.9 (4)	C9—C8—H8B	108.9
C1—N1—C3	108.5 (4)	H8A—C8—H8B	107.8
C4—N1—C3	123.8 (4)	C10—C9—C14	116.3 (5)
C1—N2—C2	108.5 (4)	C10—C9—C8	124.1 (5)
C1—N2—C8	124.7 (4)	C14—C9—C8	119.5 (5)
C2—N2—C8	126.9 (4)	C11—C10—C9	123.4 (5)
C4—N3—C7	115.0 (4)	C11—C10—Br1	116.7 (5)
C4—N3—Cu1	125.5 (3)	C9—C10—Br1	119.9 (4)
C7—N3—Cu1	119.4 (3)	C12—C11—C10	119.4 (6)
C4—N4—C5	116.2 (4)	C12—C11—H11	120.3
C1—O1—Cu1	118.2 (3)	C10—C11—H11	120.3
O1—C1—N2	126.1 (4)	C11—C12—C13	120.3 (6)
O1—C1—N1	127.3 (4)	C11—C12—H12	119.9
N2—C1—N1	106.5 (4)	C13—C12—H12	119.9
C3—C2—N2	109.7 (4)	C12—C13—C14	119.8 (6)
C3—C2—H2	125.1	C12—C13—H13	120.1
N2—C2—H2	125.1	C14—C13—H13	120.1
C2—C3—N1	106.8 (4)	C13—C14—C9	120.8 (6)
C2—C3—H3	126.6	C13—C14—H14	119.6
N1—C3—H3	126.6	C9—C14—H14	119.6
N4—C4—N3	127.3 (5)		

Symmetry code: (i) −*x*+1, −*y*+1, −*z*+1.
